# First molecular evidence of *Bartonella* spp. and hemoplasmas in cattle from Mozambique

**DOI:** 10.1007/s11250-026-04877-2

**Published:** 2026-01-28

**Authors:** Caroline Tostes Secato, Carlos António Matos, Daniel Antônio Braga Lee, Jovêncio Mateus Sada, Anna Claudia Baumel Mongruel, Eliz Oliveira Franco, Victória Valente Califre de Mello, Rosangela Zacarias Machado, Marcos Rogério André

**Affiliations:** 1 Graduate Program in Agricultural Microbiology, School of Agricultural and Veterinarian Sciences (FCAV), São Paulo State University (UNESP), Jaboticabal, SP 14884-900 Brazil; 2 Vector-Borne Bioagents Laboratory (VBBL), Department of Pathology, Reproduction and One Health, School of Agricultural and Veterinarian Sciences (FCAV), São Paulo State University (UNESP), Jaboticabal, SP 14884-900 Brazil; 3 Imunodot Diagnósticos Veterinários, Rua Dr. Mario de Campos, nº1150, Jardim São Marcos I, Jaboticabal, SP 14887-200 Brazil; 4 Direccao de Ciencias Animais, Maputo, Moçambique

**Keywords:** Hemotropic mycoplasmas, *Bartonella bovis*, *Candidatus* Mycoplasma haemobos, *Mycoplasma wenyonii*, Livestock

## Abstract

Although Mozambique is amongst the countries that suffer the most economic losses due to tick-borne pathogens in cattle, little is known about the occurrence and genetic diversity of these agents. The present study aimed to detect, through molecular methods, the occurrence of *Bartonella* spp. and hemoplasmas in cattle sampled in Maputo Province, Mozambique. Between June and July 2014, DNA samples were extracted from the blood of 222 animals and subjected to real-time PCR (qPCR) targeting the 16–23S rRNA intergenic spacer region (ITS) of *Bartonella* sp., as well as to PCR assays for ‘*Candidatus* Mycoplasma haemobos’ and *Mycoplasma wenyonii* based on the 16S rRNA gene. Samples that tested positive in the qPCR for *Bartonella* sp. were subjected to cPCR assays for characterization, targeting seven molecular markers (*gltA*, *ftsZ*, *nuoG*, *groEL*, *pap31*, *rpoB* genes, and the 16S–23S rRNA intergenic spacer - ITS). Among the 222 samples, 49 (22.1%) were positive in the qPCR for *Bartonella* spp. The *gltA*, *ftsZ*, and *nuoG* sequences obtained in the PCR assays showed > 99.5% identity to *Bartonella bovis*. The five sequences obtained from samples positive for the *ribC* gene could not be successfully sequenced. No samples tested positive in the PCR assays based on the *groEL*, *pap31*, *rpoB* genes, or the intergenic 16S-23S rRNA (ITS) region. On the other hand, 153/222 (68.9%) were positive for hemoplasmas: 29/153 (18.9%) only for *M. wenyonii*, 24/153 (15.7%) only for ‘*Ca.* M. haemobos’, and 70/153 (45.7%) for both species. Co-positivity for all three agents was detected in 11/222 (4.9%) cattle. This study reports the first molecular detection of *B. bovis*, ‘*Ca.* M. haemobos’, and *M. wenyonii* in cattle sampled in Mozambique, demonstrating that these hemopathogens are circulating in the region. Adult cattle showed higher odds of testing positive for hemoplasmas. The simultaneous detection of these agents highlights the importance of continuous epidemiological surveillance, especially because subclinical infections may compromise animal productivity and enable the silent maintenance of these pathogens within herds. These findings expand the current understanding of cattle health in Mozambique and provide essential data for developing monitoring, control, and prevention strategies tailored to local conditions. The real impact of *B. bovis* and hemoplasma infections on the productive performance of beef and dairy cattle should be further investigated.

## Introduction

Mozambique stands out among the countries that suffer the greatest economic losses due to arthropod-borne pathogens. The situation worsened in the 1990s, when initiatives aimed at improving livestock production in the region were implemented—such as cattle restocking and the importation of animals from neighboring countries—without proper health screening of these animals. This led to the spread of various tick-borne pathogens (Simuunza et al. 2011; Tembue et al. 2011). Livestock plays a significant socio-economic role for the rural population in Africa, serving not only as a source of food but also as an important source of income for many households (Observatório do Meio Rural, OMR, 2020). In 2022, Mozambique had a cattle population of over two million heads. The majority of these animals (89%) were raised by the family sector, which corresponds to subsistence farming, while the commercial sector accounted for approximately 11% (Boletim de Estatísticas Pecuárias 2012–2022).

The genus *Bartonella* (Hyphomicrobiales: Bartonellaceae) comprises Gram-negative, facultative intracellular, aerobic, and fastidious alpha-2-proteobacteria (Birtles and Raoult 1996; Birtles 2005; Kosoy et al. 2012), transmitted by hematophagous arthropods, such as fleas, flies, mosquitoes, and lice (Chomel et al. 1996; Roux and Raoult 1999). These bacteria primarily infect erythrocytes and endothelial cells, but may also parasitize dendritic cells, monocytes, and macrophages in mammals, which serve as reservoirs (Morse et al. 2012; Gutiérrez et al. 2015; Gonçalves et al. 2018, 2020). Additionally, depending on the *Bartonella* species, transmission can occur through scratches, bites, or contact with the blood or body fluids of infected animals (Birtles and Raoult 1996; Breitschwerdt 2014). To date, 42 *Bartonella* species have been described (https://lpsn.dsmz.de/genus/bartonella), of which at least 20 have zoonotic potential (Marejová et al. 2021).

Cattle can be parasitized by *Bartonella bovis*, *B. chomelii*, *B. schoenbuchensis*, *B. melophagi*, *B. henselae*, and ‘*Candidatus* Bartonella davousti’ (Gutiérrez et al. 2014; Dahmani et al. 2017; Boularias et al. 2020). Although most infected ruminants are asymptomatic, endocarditis has been reported in cattle in France (Maillard et al. 2007). The presence of *Bartonella* sp. in cattle from Africa had been reported in Algeria (15.3%) (Boularias et al. 2020), Côte d’Ivoire (20%) (Raoult et al. 2005), Senegal (27.9%) (Dahmani et al. 2017), and Somalia (3.1%) (Osman et al. 2024). *Bartonella bovis* and *‘Ca.* B. davousti’ were detected in cattle from Senegal (Dahmani et al. 2017) and *B. bovis* and *B. chomelii* were detected in cattle from Algeria (Boularias et al. 2020). Additionally, *B. bovis* has been detected in cattle from Côte d’Ivoire (Raoult et al. 2005) and Somalia (Osman et al. 2024). In Algeria, *B. bovis* and *B. chomelii* DNA was detected in 10% and 5.4% cattle, respectively (Boularias et al. 2020). In Mozambique, *Bartonella* sp. and *B. bovis* have previously been detected in African buffaloes (*Syncerus caffer*) (Gonçalves et al. 2018).

Hemoplasmas (hemotropic mycoplasmas) are pleomorphic, Gram-negative cell wall-lacking bacteria, which parasitize the surface of erythrocytes in a wide variety of animals and humans. This localization facilitates their transmission through hematophagous arthropod vectors or aggressive interactions between animals (Neimark et al. 2001; Messick 2004; Biondo et al. 2009). Two hemoplasma species are known to parasitize cattle: *Mycoplasma wenyonii* and ‘*Candidatus* Mycoplasma haemobos’ (Oren et al. 2020). In cattle, chronic infection - characterized by anemia, fever, reduced milk production, lymphadenopathy, anorexia, and depression - can result in significant economic losses (Smith et al. 1990; Messick 2004; Tagawa et al. 2008). Acute infections caused by these agents are rare in cattle (Smith et al. 1990). The presence of hemoplasmas in cattle in Africa has been reported in cattle from Uganda (32.2%) (Byamukama et al. 2020) and Nigeria (64%) (Happi et al. 2020).

Although *M. wenyonii* and ‘*Ca.* M. haemobos’ have been molecularly detected in flies (*Haematobia irritans*, *Stomoxys calcitrans*), lice (*Haematopinus eurysternus*), horseflies (*Tabanus* spp.), and ticks (*Rhipicephalus microplus*, *Haemaphysalis bispinosa*, and *Ixodes ricinus*) (Hofmann-Lehmann et al. 2004; Hornok et al. 2011; Song et al. 2013; Mohd et al. 2017; Shi et al. 2019; Stevanović et al. 2022; Shi et al. 2022), the role of arthropods in the transmission of these agents remains to be fully elucidated. Transstadial transmission of ‘*Ca*. M. haemobos’ has been demonstrated in *R. microplus* (Shi et al. 2019). Additionally, transplacental transmission of hemoplasmas in cattle has been reported in Germany, Hungary, and Brazil (Hornok et al. 2011; Girotto-Soares et al. 2016; Niethammer et al. 2018; Stadler et al. 2019).

Although *Bartonella* spp. and hemoplasmas have been molecularly detected in African buffaloes (*S. caffer*) in Mozambique (Gonçalves et al. 2018), studies on the occurrence and molecular identity of these agents in cattle in the country are lacking. This study represents the first report of the presence of hemoplasmas and *Bartonella* spp. in domestic cattle from Mozambique.

## Materials and methods

### Ethical aspects

This study was approved by the Ethics Committee for the Use of Animals at São Paulo State University “Júlio de Mesquita Filho” (UNESP) – Jaboticabal campus (protocols numbers 017259/14 and 003891/23).

### Sampled species and study areas

**Fig. 1:**
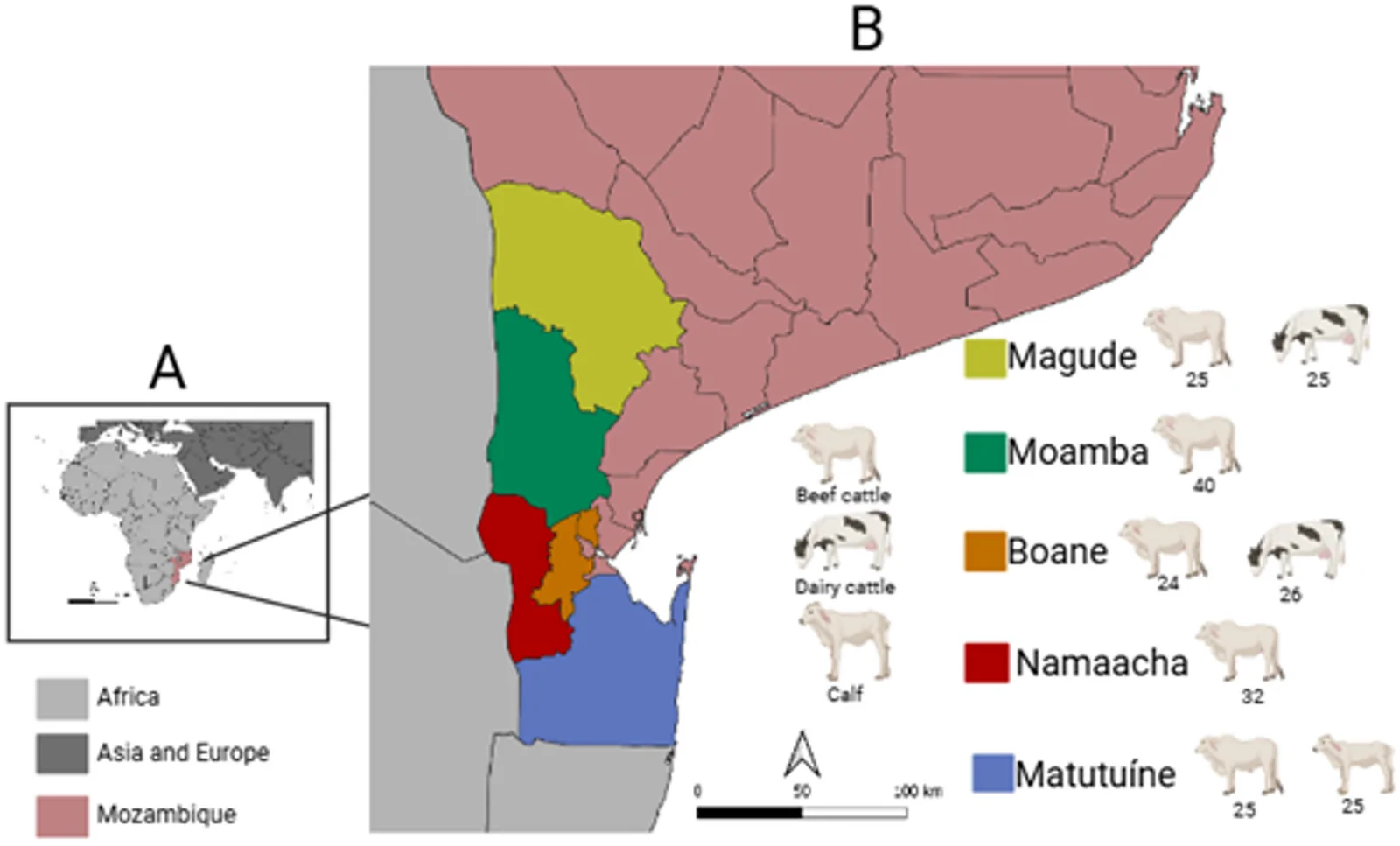
The map shows in **A**, Mozambique in pink, located within the African continent, which is displayed in light gray. The Asian and European continents are also visible. In **B**, there is a zoomed-in view of Mozambique, showing its provinces. Of these, only five are relevant to the present study and are represented by five different colors as shown on the map. Additionally, next to the name of each province, along with its color, the number of animals and their reproductive status are also indicated

In June and July 2014, 222 blood samples were collected from dairy cattle (*n* = 51) (Jersey and Friesians) in a semi-intensive farming system, and beef cattle (*n* = 171) (Nguni and Nguni crosses) in an extensive farming system across five districts. Additionally, all sampled animals showed no apparent clinical signs at the time of sampling, although they were infested with ticks and flies. Hematological and productivity parameters were not assessed in this study. Sampling was performed by convenience and included both dairy and beef cattle. The number of animals sampled may not be representative of the national herd, and therefore, the findings of the present study should not be extrapolated at a national level. Out of 222 cattle, 26 dairy cattle and 24 beef cattle were sampled in Umbeluzi (Boane district), 25 dairy cattle and 25 beef cattle at the Chobela Zootechnical Station (Magude district), 25 adult beef cattle and 25 beef calves in the Catuane region (Matutuíne district), 32 beef cattle in Matsequenha (Namaacha district), and 40 beef cattle in Josina Machel (Moamba district), Maputo, Mozambique (Fig. 1). Except for Chobela, where there was a clear separation between dairy and beef cattle, in the other sampled districts, the animals belonged to the same farm/herd, sharing pastures and a single water source. Five milliliters of blood were collected from the jugular or tail vein into EDTA-containing tubes for further molecular analysis (Fernandes et al. 2019; Matos et al. 2019).

### DNA extraction and PCR for the mammal glyceraldehyde-3-phosphate dehydrogenase (*gapdh*) gene

DNA extraction from cattle blood samples was performed using the QIAamp DNA Blood Mini Kit (Qiagen, Madison, USA), following the manufacturer’s recommendations. Concentration and 260/280 and 260/230 ratios of extracted DNA samples were measured using a spectrophotometer (Nanodrop, Thermo Scientific^®^) and then stored at -20 °C until PCR assays were conducted.

To avoid false negative results due to the presence of inhibitors, the DNA samples were subjected to conventional PCR (cPCR) aiming to amplify the endogenous gene glyceraldehyde-3-phosphate dehydrogenase (*gapdh*) from mammals, following the protocol described by Birkenheuer et al. (2003). Only positive samples in the referred PCR were subsequently subjected to PCR assays for *Bartonella* spp. and hemoplasmas.

### Quantitative real-time PCR (qPCR) and conventional PCR assays for the detection and characterization of *Bartonella* spp.

DNA samples positive in the *gapdh*-based PCR were subjected to a qPCR for *Bartonella* spp. based on the 16–23S rRNA intergenic region (ITS) (Oteo et al. 2017; Breitschwerdt et al. 2019). The real-time PCR (qPCR) assay was performed using a probe-based fluorescent chemistry (TaqMan^®^). The thermal cycling protocol consisted of an initial denaturation at 95 °C for 3 min, followed by 45 cycles of denaturation at 94 °C for 10 s, annealing at 68 °C for 10 s, and extension at 72 °C for 10 s with plate reading. A final extension step was performed at 72 °C for 30 s. Serial dilutions of G-block (IDT^®^ฏ) containing the target sequence of *Bartonella henselae* were performed to obtain standards with different target DNA concentrations (2.0 × 10⁷ to 2.0 × 10⁰ copies/µL) to be used as positive controls and standard curve construction for estimating *Bartonella* spp. copy numbers/µL. Although the synthetic G-Block fragment contained *B. henselae* ITS sequence, the primers used in the assay are genus-specific and therefore capable of detecting all known *Bartonella* species. The number of G-block copies was determined using the equation (Xg/µL DNA/[plasmid size (bp) × 660]) × 6.022 × 10²³ G-block copies/µL. All DNA samples were initially tested in duplicates. Duplicates with a Cq difference greater than 0.5 were retested in triplicates. Amplification efficiency (E) was calculated from the slope of the standard curve in each run using the following formula (E = 10^− 1/slope^) (Bustin et al. 2009). Ultrapure water (Promega^®^) was used as a negative control for all reactions.

Positive samples from the qPCR assay were subjected to PCR assays targeting the *gltA* (citrate synthase gene, which encodes the enzyme citrate synthase that plays a crucial role in the Krebs cycle) (Norman et al. 1995; Billeter et al. 2011), *ftsZ* (filamenting temperature-sensitive mutant Z, a gene that encodes the cell division protein) (Paziewska et al. 2011), *groEL* (chaperonin-encoding gene belonging to the *hsp60* family) (Paziewska et al. 2011), *rpoB* (gene responsible for encoding the β-subunit of bacterial RNA polymerase) (Renesto et al. 2001), *ribC* (gene encoding the riboflavin biosynthesis protein) (Johnson et al. 2003), *nuoG* (gene that encodes a subunit of the NADH: ubiquinone oxidoreductase complex, also known as Complex I of the bacterial respiratory chain) (Colborn et al. 2010), *pap-31* (that encodes the Pap31 protein, which is an outer membrane protein that may be involved in bacterial adhesion to host cells and its virulence) genes (Maggi and Breitschwerdt 2005a), and 16–23 S rRNA intergenic region (ITS - region is transcribed but does not code for proteins and is removed during rRNA processing) (Maggi and Breitschwerdt 2005b). Ultrapure water (Promega^®^) was used as a negative control for all reactions. A DNA sample of *Bartonella henselae* (Dias et al. 2023) was used as a positive control in all reactions.

### PCR assays for detection and molecular characterization of hemoplasmas

DNA blood samples that were positive for the endogenous gene were subjected to two PCR assays targeting the 16 S rRNA gene of ‘*Ca.* M. haemobos’ (173 bp) and *M. wenyonii* (195 bp) (Nishizawa et al. 2010). Selected positive samples were subjected to a PCR assay for hemoplasmas to amplify a 620 bp fragment of the 16S rRNA gene (Maggi et al. 2013). Cattle blood DNA samples positive for these agents (de Mello et al. 2019) were used as positive controls. Ultrapure water (Promega^®^) was used as a negative control.

### Agarose gel electrophoresis

The PCR products were subjected to horizontal electrophoresis in a 1.0% agarose gel stained with Ethidium Bromide (0.5 µL/mL) in TBE running buffer pH 8.0 (44.58 M Tris-base; 0.44 M boric acid; 12.49 mM EDTA). Electrophoresis was performed at 95 V/150 mA for 60 min. A 100 bp molecular weight marker (Life Technologies^®^) was used to determine the size of the amplified products. The results were visualized and analyzed using a UV light transilluminator, coupled with a data analysis software (ChemiDoc MP Imaging System, BIO RAD^®^).

### Purification and sequencing of amplified products

Amplicons were purified using the Wizard^®^ DNA Clean-Up System (Promega^®^), according to the manufacturer’s recommendations. The sequencing of the amplified products was performed bidirectionally using an automated technique based on the chain termination method with dideoxynucleotides (Sanger et al. 1977), employing the same primers used in the PCR assays. Sequencing was conducted on the ABI PRISM 3700 DNA Analyzer (Applied Biosystems) at the Biological Resources and Genomic Biology Center (CREBIO), located in the Department of Technology at the Faculty of Agricultural and Veterinary Sciences - FCAV/UNESP Jaboticabal.

### Sequence analysis and phylogenetic tree construction

The analysis of the electropherograms generated from the sequencing and the construction of consensus sequences were performed using the Phred-Phrap program version 23 (Ewing et al. 1998; Ewing and Green 1998), with a minimum phred quality value of 20 for the bases. The BLASTn program (Altschul et al. 1990) was used to compare the obtained sequences with those previously deposited in GenBank (http://www.ncbi.nlm.nih.gov/genbank). The sequences were saved in FASTA format and aligned with other sequences of the same gene retrieved from the GenBank database using the MAFFT software (Katoh et al. 2002). The alignments saved in FASTA format were converted to Nexus format using the Alignment Transformation Environment software (Glez-Peña et al. 2010). Maximum likelihood analyses were performed using the IQtree cluster (Nguyen et al. 2015). The evolutionary model was determined using the jModelTest 2 software (Guindon et al. 2003; Darriba et al. 2012). Bootstrap analyses (Felsenstein 1985) with 1000 repetitions were used to evaluate the support of the clades for maximum likelihood analyses. The editing of the phylogenetic trees and rooting (via an outgroup) was done using the Treegraph 2.0.56–381 beta software (Stover and Muller 2010).

### Statistical analysis

To assess the association between the positivity for ‘*Ca.* M. haemobos’, *M. wenyonii*, and *Bartonella* spp. with the binary variables analyzed (age; beef/dairy), Fisher’s Exact Test and the Chi-square test with Yates correction were used, according to the distribution of expected frequencies. To analyze the association of the variables with the occurrence of co-infection, co-infection was considered as positivity for hemoplasmas (‘*Ca.* M. haemobos’ and/or *M. wenyonii*) and *Bartonella* spp. in the same sample.

To analyze the association between positivity and locality, the Chi-square test of independence was used. Additionally, it was investigated whether positivity for hemoplasmas (‘*Ca.* M. haemobos’ and/or *M. wenyonii*) could influence the occurrence of *Bartonella* spp. in the sampled animals using the Chi-square test with Yates correction.

Additionally, the prevalence for each agent was calculated. To verify whether the difference in proportions of animals positive for *Bartonella* spp. and hemoplasmas in the sampled population was significant, the McNemar test was performed. All analyses were conducted using RStudio v 4.2.3 and OpenEpi v.3.

## Results

### Endogenous control of PCR

All 222 cattle blood DNA samples were positive in the PCR targeting the amplification of the mammalian endogenous *gapdh* gene.

### Positivity and phylogenetic inferences for *Bartonella* spp.

Among the 222 DNA samples, 49 (22.1%) (CI: 17.12–27.98%) were positive in the qPCR for *Bartonella* spp. based on the 16–23 S rRNA intergenic region (ITS). The positivity for *Bartonella* spp. according to locality and production type (beef vs. dairy) is presented in Table 1. The absolute quantification of *Bartonella* DNA ranged from 9.47 × 10^− 1^ to 1.48 × 10^4^ copies/µL. The average efficiency of the qPCR reactions was *E* = 105.3%, slope = -3.197, *r²* = 0.917 and y-intercept = 35.510.

**Table 1:** Positivity of *Bartonella* spp., *‘Candidatus* Mycoplasma haemobos’, and *Mycoplasma wenyonii*, and coinfections with all three agents according to locality and production type

Location	Beef cattle (*n* = 171)				Dairy cattle (*n* = 51)			
	*Bartonella* spp. (*n* = 38)	*‘Ca.* M. haemobos’ (*n* = 81)	*M. wenyonii* (*n* = 85)	Coinfection	*Bartonella* spp. (*n* = 11)	*‘Ca.* M. haemobos’ (*n* = 33)	*M. wenyonii* (*n* = 35)	Coinfection
Umbeluzi	9/24 (37.5%)	10/24 (41.7%)	19/24 (79.2%)	1/24 (4.2%)	8/26 (30.8%)	20/26 (76.9%)	22/26 (84.6%)	7/26 (26.9%)
Estação Zootécnica de Chobela	9/25 (36%)	7/25 (28%)	14/25 (56%)	3/25 (12%)	3/25 (12%)	13/25 (52%)	13/25 (52%)	0/25 (0%)
Namaacha	4/32 (12.5%)	13/32 (40.6%)	15/32 (46.9%)	0/32 (0%)	0/0	0/0	0/0	0/0
Catuane	4/25 adults (16%); 9/25 calves (36%)	15/25 adults (60%); 4/25 calves (16%)	14/25 adults (56%); 1/25 calves (4%)	0/50 (0%)	0/0	0/0	0/0	0/0
Moamba	3/40 (7.5%)	32/40 (80%)	22/40 (55%)	0/40 (0%)	0/0	0/0	0/0	0/0
Total	38/171 (22.2%)	81/171 (47.4%)	85/171 (49.7%)	4/171 (2.3%)	11/51 (21.6%)	33/51 (64.7%)	35/51 (68.6%)	7/51 (13.7%)

Of these 49 samples, five (10.2%) were positive in the PCR assays targeting the *gltA* gene, four (8.2%) for the *nuoG* gene, five (10.2%) for the *ribC* gene, and three (6.1%) for the *ftsZ* gene. No samples tested positive in the PCR assays based on the *groEL*, *pap31*, *rpoB* genes, or the intergenic 16–23 S rRNA (ITS) region. Three readable sequences were obtained for the *ftsZ* gene (one from the Umbeluzi district, one from Moamba, and one from Chobela), two for the *gltA* gene (one from the Umbeluzi district, and one from Chobela), and one sequence for the *nuoG* gene (from the Umbeluzi district), all showing > 99.5% identity with *B. bovis* (Table 2).

**Table 2:** BLASTn results of *ftsZ*, *gltA*, And *NuoG* gene sequences of *Bartonella* spp. And the 16 S rRNA gene sequences of hemoplasmas detected in blood samples from cattle in Mozambique

Gene	Size	Animal-clone ID	Query cover	E-value	Genbank data	Host/Country
*ftsZ*	480 bp	Mo8 – cattle (PV440602)	100%	0.0	99.58% *B. bovis* (KF193409)	Cattle/Guatemala
*ftsZ*	441 bp	Ch18 – cattle (PV440603)	100%	0.0	99.54% *B. bovis* (KF193409)	Cattle/Guatemala
*ftsZ*	479 bp	U39 – cattle (PV440604)	100%	0.0	99.79% *B. bovis* (KF193409)	Cattle/Guatemala
*gltA*	492 bp	U39 – cattle (PV440605)	100%	0.0	99.60% *B. bovis* (KF199895)	Cattle/France
*gltA*	502 bp	Ch33 – cattle (PV440606)	100%	0.0	99.79% *B. bovis* (KF199895)	Cattle/France
*nuoG*	372 bp	U39 – cattle (PV440601)	100%	0.0	99.79% *B. bovis* (EF659938)	Cattle/Thailand
16 S rRNA	592 bp	U14 – cattle (PV590298)	100%	0.0	100% *M. wenyonii* (EF221880.1)	Cattle/China
16 S rRNA	569 bp	U34 – cattle (PV590300)	100%	0.0	100% *‘Ca.* M. haemobos’ (EU367965.1)	Cattle/Japan

**Fig. 2:**
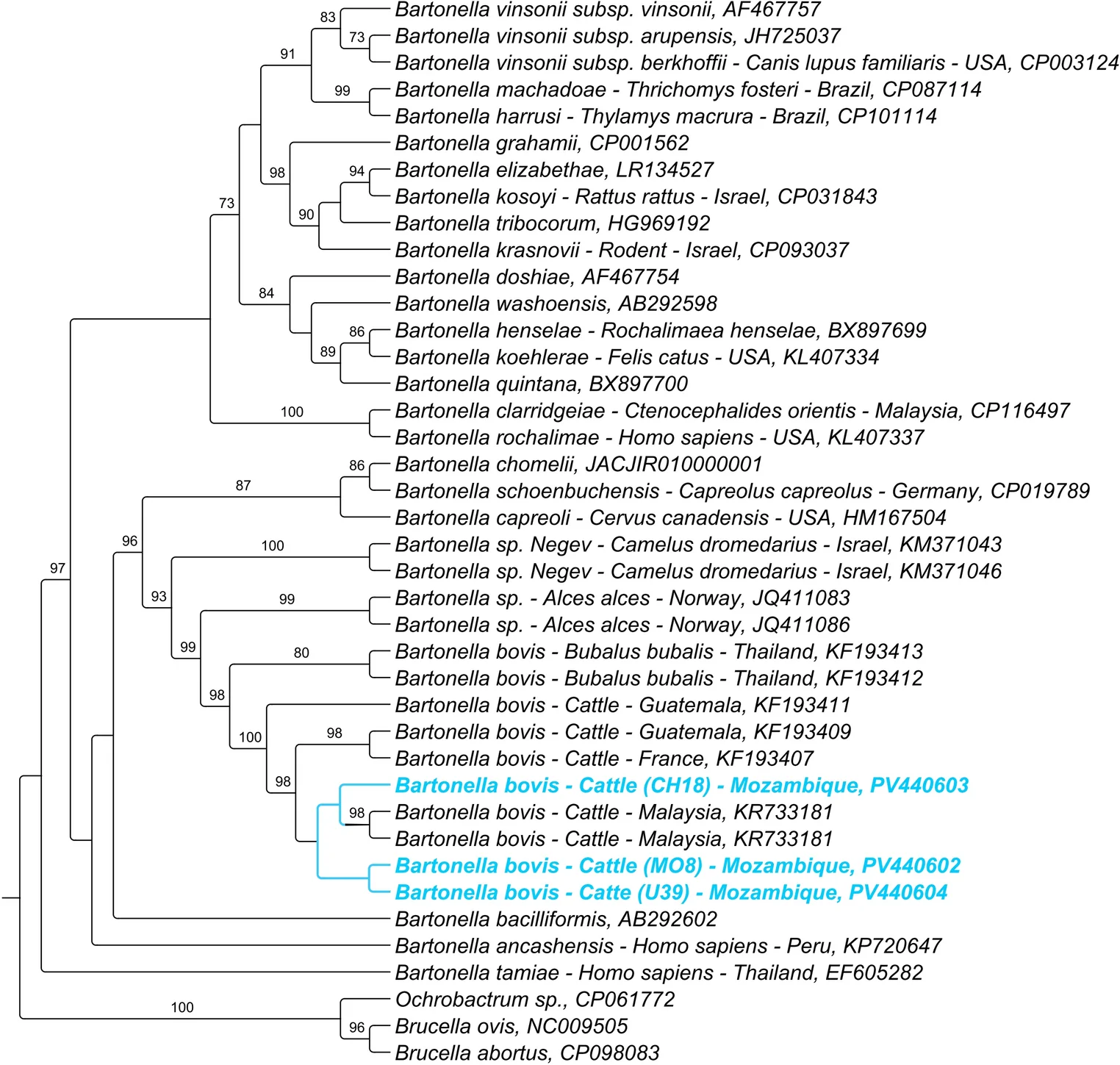
Phylogenetic tree based on a sequence alignment of the *ftsZ* gene (~ 473 bp) of *Bartonella* spp. using the Maximum Likelihood (ML) method and GTR + G as the evolutionary model. Only bootstrap values > 70 were shown on the tree. The sequences detected in the present study are highlighted in light blue. *Ochrobactrum sp.* (CP061772), *Brucella ovis* (NC009505), and *Brucella abortus* (CP098083) were used as an outgroup

The phylogenetic analysis based on the *ftsZ* gene of *Bartonella* spp. grouped the two sequences obtained in the present study with sequences of *B. bovis* from cattle in Malaysia **(**Fig. 2**).**

**Fig. 3:**
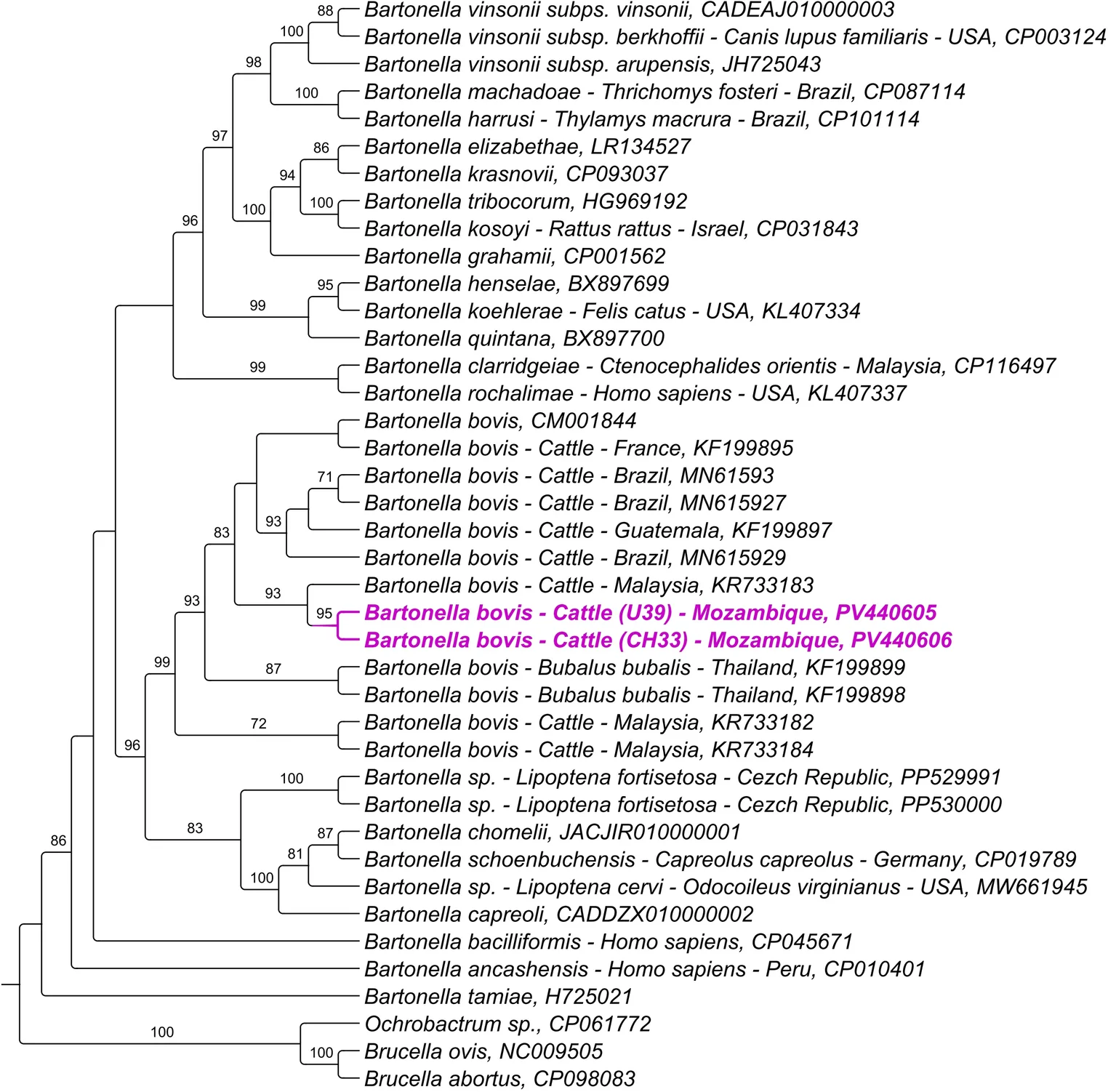
Phylogenetic tree based on an alignment of *Bartonella* spp. *gltA* gene sequences (~ 531 bp) using the Maximum Likelihood (ML) method and GTR + I + G as the evolutionary model. Only bootstrap values > 70 were shown on the tree. The sequences detected in the present study are highlighted in purple. *Ochrobactrum sp.* (CP061772), *Brucella ovis* (NC009505), and *Brucella abortus* (CP098083) were used as an outgroup

The phylogenetic analysis based on the *gltA* gene of *Bartonella* spp. grouped the two sequences obtained in the present study with *B. bovis* sequences obtained from cattle in Malaysia (Fig. 3).

**Fig. 4:**
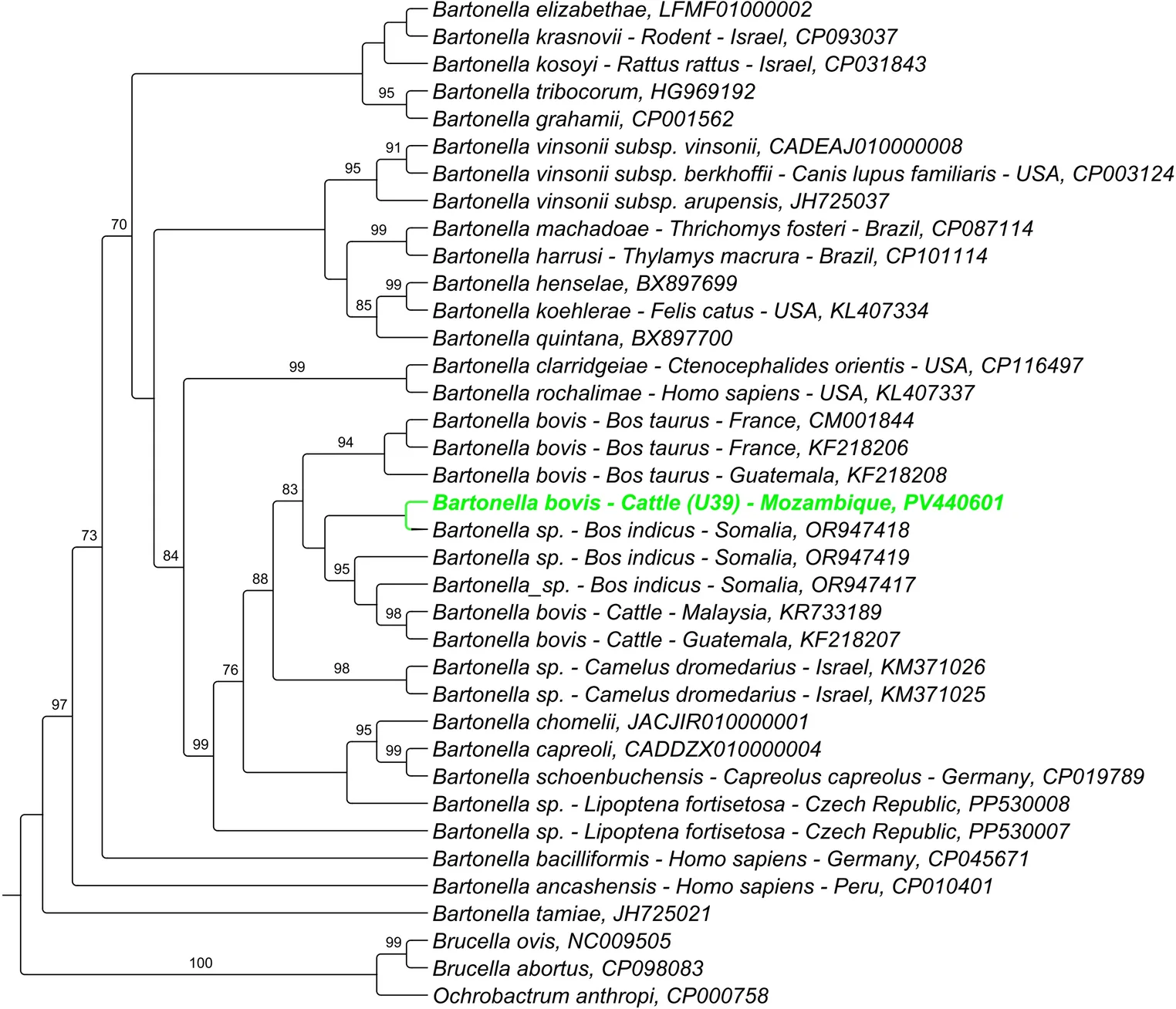
Phylogenetic tree based on a sequence alignment of the *nuoG* gene (~ 365 bp) for *Bartonella* spp. using the Maximum Likelihood (ML) method and GTR + I + G as the evolutionary model. Only bootstrap values > 70 were shown on the tree. The sequences detected in the present study are highlighted in green. *Ochrobactrum anthropi* (CP000758), *Brucella ovis* (NC009505), and *Brucella abortus* (CP098083) were used as an outgroup

The phylogenetic analysis based on the *nuoG* gene of *Bartonella* spp. grouped the sequence obtained in the present study with sequences of *Bartonella* sp. from *Bos indicus* from Somalia and sequences of *B. bovis* obtained from cattle from Malaysia and Guatemala (Fig. 4).

### Positivity and phylogenetic inferences for hemoplasmas

Out of the 222 cattle blood DNA samples, 153 (68.9%) (CI: 62.55–74.64%) were positive for hemoplasmas: 18.9% (29/153) were positive only for *M. wenyonii*, 15.7% (24/153) only for ‘*Ca.* M. haemobos’, and 45.7% for both hemoplasma species. The positivity for ‘*Ca.* M. haemobos’ and *M. wenyonii* according to locality and productive aptitude (beef vs. dairy) are presented in Table 1.

The two 16 S rRNA sequences (~ 620 bp; both from the Umbeluzi district) obtained showed 100% identity with ‘*Ca.* M. haemobos’ from cattle from Japan and *M. wenyonii* from cattle from China (Table 2).

**Fig. 5:**
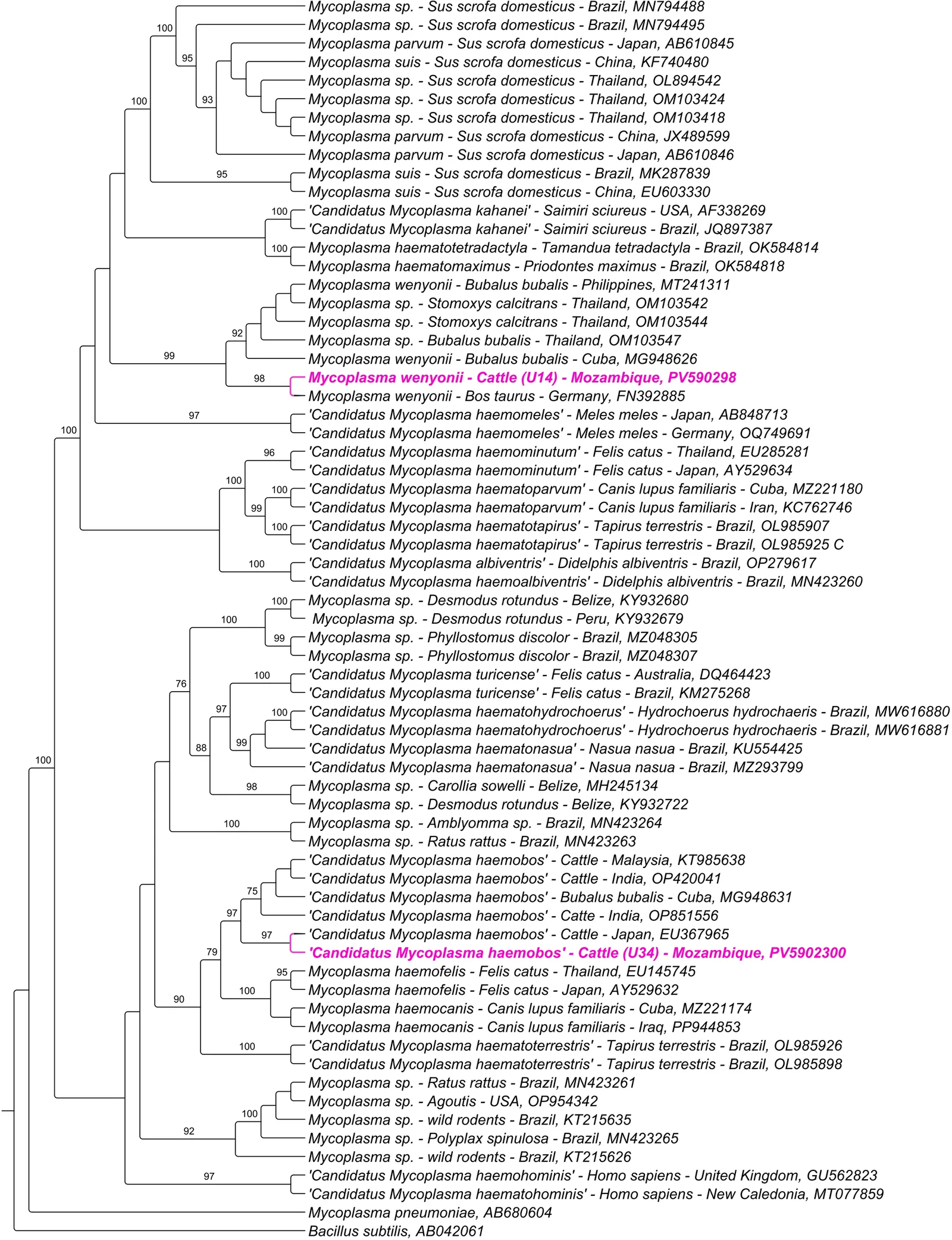
Phylogenetic tree based on an alignment of 16 S rRNA gene sequences (~ 605 bp) of hemotropic *Mycoplasma* spp. using the Maximum Likelihood (ML) method and GTR + I + G as the evolutionary model. Only bootstrap values > 70 were shown on the tree. The sequences detected in the present study are highlighted in pink. *Mycoplasma pneumoniae* (AB680604) and *Bacillus subtilis* (AB042061) were used as an outgroup

The phylogenetic analysis based on the 16 S rRNA gene of *Mycoplasma* spp. grouped one of the sequences obtained in this study with ‘*Ca.* M. haemobos’ from Japan. The other sequence grouped with *M. wenyonii* detected in cattle from Germany (Fig. 5).

### Association between positivity and productive aptitude/breed

The statistical analysis showed that dairy cattle appeared to be at least twice as likely to test positive for hemoplasmas compared to beef cattle. However, no significant association was found between productive aptitude or breed and positivity for *Bartonella* spp. or co-infection with hemoplasmas (Table 3).

**Table 3:** Analysis of the association between positivity for the agents and the productive aptitude of the animals tested

Agent	Test	+/*n* (%)	*p*-value (2-tails)	OR (IC)
‘*Candidatus* Mycoplasma haemobos’	Chi-square test with Yates’ correction	Dairy: 33/51 (64.7%); Beef: 81/171 (47.4%)	**0.04396**	2.037 (1.066–3.894)
*Mycoplasma wenyonii*	Chi-square test with Yates’ correction	Dairy: 35/51 (68.6%); Beef: 85/171 (49.7%)	**0.02646**	2.213 (1.14–4.295)
*Bartonella* spp.	Chi-square test with Yates’ correction	Dairy: 11/51 (21.6%); Beef: 38/171 (22.2%)	0.9254	1.039 (0.486–2.218)
Coinfection	Chi-square test with Yates’ correction	Dairy: 7/51 (13.7%); Beef: 23/171 (13.4%)	0.8549	1.024 (0.411–2.544)

+ = positive animals; n = total number of animals tested in the category; OR = Odds ratio; CI = 95% confidence interval. The *p*-value was considered significant when < 0.05

### Association between positivity and age

The statistical analysis showed that infection by *‘Ca.* M. haemobos*’* and *M. wenyonii* appeared to be higher in adults when compared to calves, but age was not associated with *Bartonella* spp. positivity or co-infection (Table 4).

**Table 4:** Analysis of the association between positivity for the agents and the category of the tested animals

Agent	Test	+/*n* (%)	*p*-value (2-tails)	OR (IC)
‘*Candidatus* Mycoplasma haemobos’	Chi-square test with Yates’ correction	Calves: 4/25 (16%); Adults: 110/197 (55.8%)	**0.0003**	6.638 (2.197–20.5)
*Mycoplasma wenyonii*	Chi-square test with Yates’ correction	Calves: 1/25 (4%); Adults: 119/197 (60.4%)	**< 0.0001**	36.62 (4.855–276.2)
*Bartonella* spp.	Chi-square test with Yates’ correction	Calves: 9/25 (36%); Adults: 40/197 (20.3%)	0.1269	2.3208 (0.9091–5.362)
Coinfection	Fisher’s Exact Test	Calves: 4/25 (16%); Adults: 26/197 (13.2%)	0.8923	1.253 (0.398–3.941)

+ = positive animals; n = total number of animals tested in the category; OR = Odds ratio; CI = 95% confidence interval. The *p*-value was considered significant when < 0.05

### Association between locality and positivity

Statistical analysis revealed a significant association between locality and positivity for all tested agents. Moamba appeared to have the highest proportion of positive animals for *‘Ca. M. haemobos’* (80%), while Umbeluzi appeared to have the highest rates for *M. wenyonii* (82%), *Bartonella* spp. (34%), and co-infections (16%). Table 1 shows the proportions of animals positive for each agent and those presenting co-infection among the three agents, according to locality.

### Analysis of the prevalence for *Bartonella* spp. and hemoplasmas

McNemar’s analysis revealed that the difference observed between the prevalence rates for *Bartonella* spp. and hemoplasmas was statistically significant, with a higher proportion of animals positive for hemoplasmas compared to *Bartonella* spp.

### Association between positivity for *Bartonella* spp. and hemoplasmas

When evaluating whether positivity for hemoplasmas could influence the occurrence of *Bartonella* spp. in the tested animals, the association analysis showed no statistically significant association (*p* > 0.05) (Table 5).

**Table 5:** Analysis of the association between positivity for hemoplasmas and positivity for *Bartonella* spp

	Positividy for Bartonella spp.			
		+/*n* (%)	OR (IC)	*p*-value (2 tails)
Positivity for hemoplasmas	Yes No	30/153 (19.6%) 19/69 (27.5%)	1.558 (0.8036-3.02)	0.2537

+ = animals positive for *Bartonella* spp.; n = total number of animals tested in the category (positive or negative for hemoplasmas); OR = Odds ratio; CI = 95% confidence interval. A *p*-value < 0.05 was considered statistically significant

### Co-positivity for *Bartonella* spp. and hemoplasmas

**Fig. 6:**
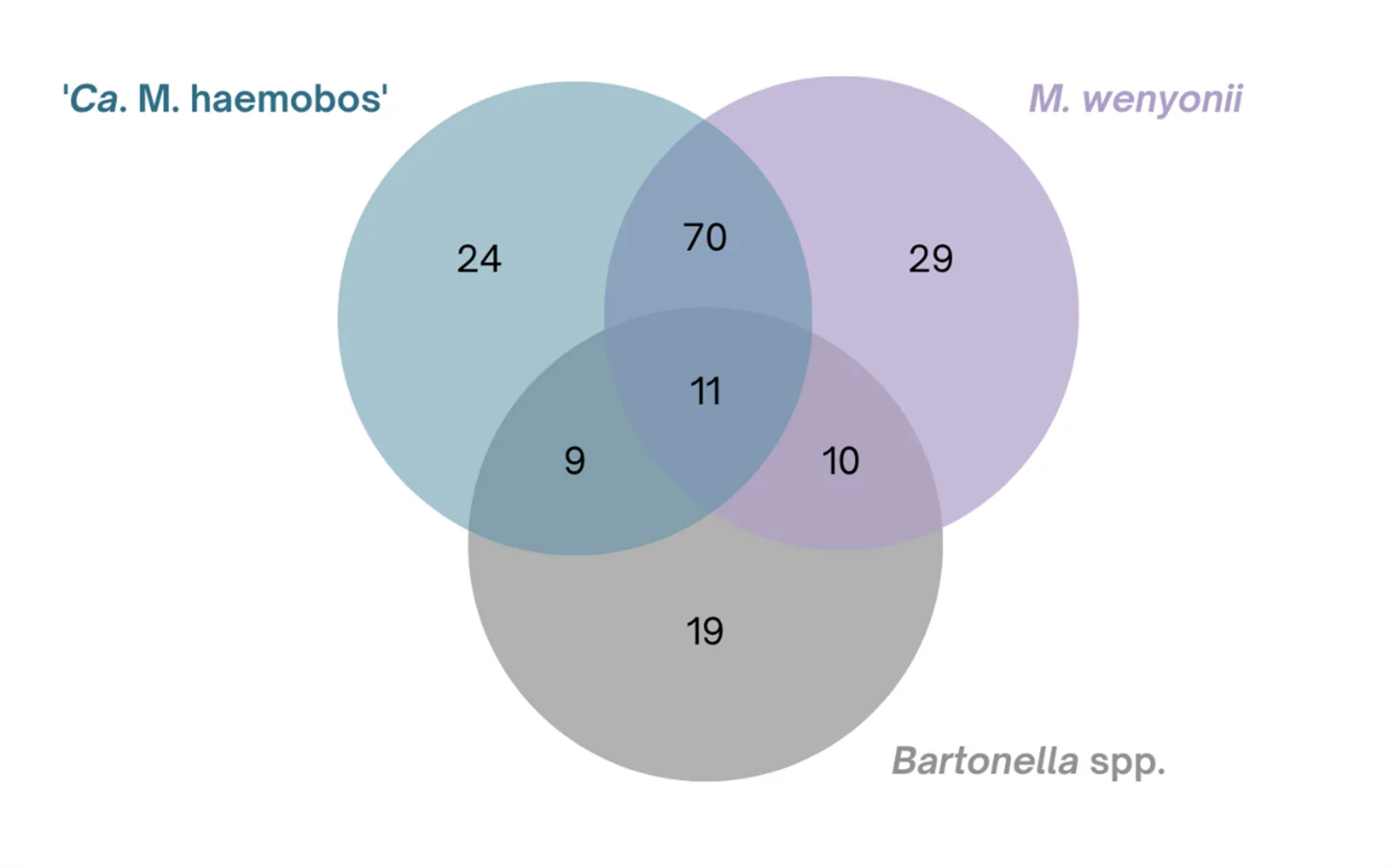
Venn diagram representing the number of animals testing positive for each of the agents studied

The present study detected co-positivity for *Bartonella* spp., ‘*Ca.* M. haemobos’, and *M. wenyonii* in 11 cattle [11/222; (4.9%)], of which seven dairy cattle (63.6%) and four beef cattle (36.4%). Co-positivity for both hemoplasma species was found in 70/153 animals (45.7%), with 48 (68.6%) being beef cattle and 22 (31.4%) dairy cattle. Co-positivity for *Bartonella* spp. and ‘*Ca.* M. haemobos’ was observed in 9/171 beef cattle (5.3%), while co-positivity between *Bartonella* spp. and *M. wenyonii* was found in 10/171 beef cattle (5.8%). A Venn diagram (Fig. 6) was constructed to illustrate the prevalence of positive animals and the observed co-infections.

## Discussion

In the present study, *Bartonella* sp. DNA was detected in 22.1% of cattle blood DNA samples from Mozambique. Until now, the presence of *Bartonella* sp. in cattle in Africa had only been reported in Algeria (15.3%) (Boularias et al. 2020), Côte d’Ivoire (20%) (Raoult et al. 2005), Senegal (27.9%) (Dahmani et al. 2017), and Somalia (3.1%) (Osman et al. 2024). On the other hand, despite substantial sampling (*n* = 221), *Bartonella* spp. was not detected in cattle from Kenya (Bai et al. 2013). The observed variation in the prevalence of *Bartonella* spp. in cattle across different countries may result from multiple factors, such as the level of infestation by putative arthropod vectors, environmental features, and the sensitivity of the molecular assay used for screening (Billeter et al. 2008; Regier et al. 2016).

*Bartonella bovis* was the only species detected in the animals sampled in the present study, corroborating the results reported by Osman et al. (2024) in Somalia. On the other hand, *B. bovis* and *‘Candidatus* B. davousti’ were detected in cattle from Senegal (Dahmani et al. 2017)d *bovis* and *B. chomelii* were detected in cattle from Algeria (Boularias et al. 2020) and Spain (Antequera-Gómez et al. 2015). Additionally, *B. bovis* has been detected in cattle in Mexico (Raya et al. 2018), Côte d’Ivoire (Raoult et al. 2005), and Brazil (Gonçalves et al. 2020). Altogether, these studies demonstrate that *B. bovis* is the main *Bartonella* spp. species detected in cattle across geographic regions. In Mozambique, *Bartonella* sp. and *B. bovis* have previously been detected in African buffaloes (*Syncerus caffer*) (Gonçalves et al. 2018). Since *B. bovis* has already been detected in a blood sample from a veterinarian in Mexico who worked on a cattle farm (Gamboa-Prieto et al. 2023), the zoonotic potential of this agent should not be overlooked.

Although 49 cattle blood samples were positive in the qPCR for *Bartonella* spp., only six readable sequences were obtained. This finding can be explained by the fact that conventional PCR is less sensitive when compared to qPCR. Another factor that might explain these findings is the level of *Bartonella* spp. bacteremia in the sampled animals. Due to the high sensitivity of qPCR, even low levels of bacteremia can be detected. However, if bacteremia is low, conventional PCR assays may fail to amplify the pathogen’s DNA, which could explain the lower number of positive results and sequences obtained in this study. Nevertheless, phylogenetic inferences performed were based on three distinct molecular markers (*ftsZ*,* gltA*, and *nuoG*) and corroborated to each other, positioning the obtained sequences within the *B. bovis* clade.

The molecular occurrence of hemoplasmas found in the present study (68.9%) was higher than that reported in cattle from Uganda (32.2%) (Byamukama et al. 2020), Nigeria (64%) (Happi et al. 2020), Turkey (31.64%) (Erol et al. 2023), Kyrgyzstan (20.94%) (Altai et al. 2023), India (43.04%) (Murugesan et al. 2024), and Korea (34%) (Kim et al. 2024). The prevalence of hemoplasma infections varies widely around the world, being mainly influenced by environmental factors (geographic distribution and climate), hosts (depending on host susceptibility, with some being more susceptible while others are more resistant), the presence of arthropod vectors (the presence and density of ticks, flies, and other arthropod vectors directly influence the spread of hemoplasmas), and management practices (Happi et al. 2020).

The statistical analysis showed that infection by *‘Ca.* M. haemobos*’* and *M. wenyonii* appeared to be higher in adults when compared to calves. These findings are consistent with those reported by Díaz-Sánchez et al. (2019) in Cuba, where dairy cattle and buffaloes (*Bubalus bubalis*) aged one year or older had a greater risk of hemoplasma infection than those under one year of age. In Japan, Tagawa et al. (2012) observed a higher prevalence of *M. wenyonii* infection in beef cattle aged one to three years compared to younger animals. Similarly, in Brazil, Girotto et al. (2012) found that female dairy cattle over two years old showed a higher prevalence of *‘Ca. M. haemobos’*. These findings were also reported by Lorusso et al. (2016) and Happi et al. (2020) in beef cattle in Nigeria. This increased prevalence for hemoplasmas in older animals could be explained by prolonged exposure to potential arthropod vectors over the course of their lives (Tagawa et al. 2012; Happi et al. 2020).

Although dairy cattle appeared to have higher chances of hemoplasma infection compared to beef cattle, it is important to consider that all dairy cattle were adults, while the sampled beef cattle were either calves or adults. As mentioned earlier, adult animals had higher chances of testing positive for hemoplasmas compared to calves. Therefore, this confounding factor cannot be ruled out as dairy cattle might have had a higher risk of hemoplasma infection simply because they were adults. Reduced milk production, abortion, and delayed estrus were reported during the acute phase of hemoplasma infection in Holstein heifers (Smith et al. 1990). Additionally, lower milk production and lower birth weight of calves were observed in Holstein dairy cows with chronic hemoplasma infection (Tagawa et al. 2013).

Cattle from Umbeluzi appeared to have the highest proportion of positive animals for *M. wenyonii*,* Bartonella* spp., and co-infection with hemoplasmas and *Bartonella* spp. On the other hand, Moamba appeared to have the highest proportion of animals positive for ‘*Ca.* M. haemobos’. Although the possible factors involved in the differences in positivity rates for hemoplasmas and *Bartonella* spp. in cattle from the different localities and production aptitudes studied are unknown, these factors may include the presence of arthropod vectors and differences in management practices on the sampled farms (Díaz-Sánchez et al. 2019; Arendt et al. 2024).

Cattle farming plays an important role in the rural economy of Mozambique, serving as an essential source of income, food, and draft power for many families (Governo de Moçambique 2019; FAO, 2020). Most of the country’s herd is raised under extensive systems, mainly in the central and southern regions, where livestock is kept in open areas with little to no sanitary or zootechnical infrastructure (Penrith et al. 2007). Although livestock production holds significant importance in Mozambique, productivity is still limited by several factors, with arthropod-borne diseases—especially those transmitted by ticks—standing out (Dahmani et al. 2017). Tick infestation are among the main health challenges faced by cattle farming in Mozambique (Kamani et al. 2010). For instance, *Rhipicephalus (Boophilus) microplus*, *Amblyomma variegatum*, and *Rhipicephalus appendiculatus* are frequently found in various regions of the country and may act as vectors of important bovine hemoparasitosis, including babesiosis, bartonellosis, hemoplasmosis, anaplasmosis, and theileriosis (Gonçalves et al. 2018; Boularias et al. 2020; Byamukama et al. 2020). Acute infections caused by these diseases are rare, but chronic infections can be characterized by anemia, fever, reduced milk production, lymphadenopathy, anorexia, and depression (OIE 2022). These infections can lead to economic losses, both due to the cost of animal treatment and the loss of livestock products, such as meat, milk, and leather (Kivaria 2006; Jonsson 2006).

In addition to ticks, other arthropod vectors are also present in cattle farming areas in Mozambique, such as blood-feeding dipterans of the genera *Stomoxys*,* Haematobia* and *Tabanus* (Mihok 2002; Baldacchino et al. 2013). Although *Bartonella* spp. and/or hemoplasmas DNA were detected in those dipterans, the real role of these insects in the transmission of such bacteria among cattle should be further investigated (Hornok et al. 2011; Deshuillers et al. 2014; Gutiérrez et al. 2015; Thongmeesee et al. 2022). The presence of these vectors is mainly associated with environmental factors, such as humidity, vegetation, and the presence of flooded or waterlogged areas, as well as the lack of effective control measures and inadequate management practices (Estrada-Penã, 2006). The country’s tropical climate favors the life cycle of these arthropods, allowing them to remain present throughout the entire year (Mapiye et al. 2009). In addition, inadequate management practices are commonly observed: many farms lack proper cattle handling facilities, such as corrals, animal restraint structures, and designated treatment areas; in some properties, the use of acaricides is irregular or even absent; the lack of rotational grazing favors the persistence of tick life cycles in pastures; there is a lack of surveillance programs aimed at the early detection and treatment of parasitic infections; furthermore, most farmers have little or no access to veterinary assistance or training programs focused on good sanitary practices and integrated parasite management (Mapiye et al. 2009; FAO et al. 2018). These factors affect not only the prevalence of diseases but also herd productivity and the potential for pathogen dissemination (Kivaria et al. 2006).

Herein, the highest positivity rate for hemoplasmas compared to *Bartonella* spp. was found among the animals sampled in the present study. The analysis of the association between positivity for hemoplasmas and *Bartonella* spp. did not show statistical significance, indicating that the presence of one agent was not associated with the occurrence of the other. Most likely, the transmission routes differ for the two groups of bacteria studied. Although transplacental transmission of hemoplasmas has been reported in cattle (Girotto-Soares et al. 2016; Niethammer et al. 2018; Stadler et al. 2019), there is no evidence in the literature regarding vertical transmission of *B. bovis* (Chastant-Maillard et al. 2015). Another hypothesis is that distinct arthropod vectors are involved in the transmission cycles of the two bacteria groups studied here. Although DNA of cattle-associated *Bartonella* spp. (Chung et al. 2004; Tsai et al. 2011; Kho et al. 2015; Boularias et al. 2020) and hemoplasmas (Hofmann-Lehmann et al. 2004; Song et al. 2013; Stevanović et al. 2022) has already been detected molecularly in blood-sucking flies and ticks, the role of arthropod vectors in the transmission of these agents requires further studies. Vector competence studies should be conducted to determine whether these arthropods are involved in the biological transmission of these agents. In addition, future studies aiming at assessing the score of tick and fly infestation in cattle are needed to check the possible association between ectoparasite burden and positivity for hemoplasmas and *Bartonella* spp. in Mozambique.

Although little is known about the pathogenesis of *M. wenyonii* and ‘*Ca.* M haemobos’, some studies suggest that the latter is more pathogenic than the former (Tagawa et al. 2010). More severe clinical signs of hemoplasmosis are expected in the presence of co-infections with both hemoplasma species or with other pathogens (Meli et al. 2010). In the present study, 45.7% of the animals had co-infection with both hemoplasma species, and 4.9% of the animals had co-infection with *Bartonella* spp., ‘*Ca.* M. haemobos’, and *M. wenyonii*. Co-infection of hemoplasmas with other hemoparasites (e.g., piroplasms, trypanosomatids, and *Anaplasma/Ehrlichia*) has already been reported in Africa (Happi et al. 2020).

One of the limitations of the present study is that the risk factors associated with *Bartonella* spp. and hemoplasma infections in beef and dairy cattle from different farms were not investigated. Additionally, ectoparasites from the sampled animals were neither collected nor quantified, which could have contributed to a better understanding of the potential vectors involved in the transmission of the agents studied in this work. Another point to consider among the limitations is that all dairy animals were adult females. This association limits the conclusions regarding the risk of infections depending on the productive aptitude/breed. The present study conducted multiple association tests without statistical correction. This poses a risk of Type I error, also known as alpha error or false positive, which occurs when a statistically significant effect or difference is concluded but, in reality, the observed results happened by chance or due to other factors unrelated to the effect being investigated.

Another limitation of the present study was the lack of evaluation of hematological changes and productivity parameters in the infected animals. Regarding the pathogenic potential of hemoplasmas, *‘Ca. M. haemobos’* and *M. wenyonii* have been detected in aborted fetuses (Hornok et al. 2011; Girotto-Soares et al. 2016; Žilić et al. 2025), highlighting the role of these pathogens in abortions. Hemoplasmas have been molecularly detected in cows with reproductive disorders, demonstrating the pathogen’s potential to cause such complications (Ferreira & Ruegg, 2024). During an outbreak of bovine anaplasmosis in Switzerland, cattle with fatal anemia were more frequently infected with *M. wenyonii* and exhibited higher *M. wenyonii* loads in the blood than animals with unrelated diseases and healthy animals. According to the authors, coinfection with *A. marginale* may increase the pathogenicity and clinical significance of bovine hemoplasmosis (Meli et al. 2010). Secondary infection with *M. wenyonii* was considered responsible for severe anemia in a cow presenting chronic bacterial pneumonia in the United States (Genova et al. 2011). Lower values of mean cell volume (MHC) and mean cell hemoglobin (MCH) and higher values of white blood cells (WBC) were observed in *‘Ca.* M. haemobos’-infected cows from Germany. *Mycoplasma wenyonii*-positive cows showed lower MCH. Animals co-infected with *M. wenyonii* and *‘Ca.* M. haemobos’ showed only higher WBC (Niethammer et al. 2018). In France, cattle positive for *M. wenyonii* presented anemia, edema and milk drop (Nouvel et al. 2019). In addition to these findings, cattle infected with hemoplasmas may also exhibit decreased weight loss, transient fever, rough hair coat, vaginal bleeding, jaundice, and reduced birth weight of infected calves (Smith et al. 1990; Tagawa et al. 2010; Genova et al. 2011; Hoelzle et al. 2011; Baggenstos et al. 2012; Tagawa et al. 2013; Gladden et al. 2016).

On the other hand, the real significance of *Bartonella* infections on cattle health is unknown. For instance, *B. bovis* was detected in a dead pregnant 7-year-old Angus cow with the aortic valve expanded by moderate fibrous connective tissue and an acidophilic thrombus in the USA (Erol et al. 2013). Maillard et al. (2007) detected *B. bovis* in 2/22 cases of endocarditis in adult cows in France.

It is noteworthy that the blood samples from cattle in Mozambique were collected in 2014 and that animals were selected by convenience; therefore, the results should be interpreted as historical reference data rather than estimates of the current prevalence of the investigated agents. This sampling strategy limits the generalizability of the findings and may not accurately reflect the present epidemiological situation, as substantial changes may have occurred over the past decade in environmental conditions, vector ecology and distribution, as well as in cattle management, population dynamics, and health or diagnostic practices. These factors can markedly influence the transmission dynamics of infectious agents, underscoring the need for caution when extrapolating the results to current settings, while also highlighting the value of this study as a baseline for future comparative investigations.

Furthermore, in the present study, only a subset of the qPCR-positive samples yielded readable sequences. Therefore, the identification of *B. bovis* at the species level applies only to the sequenced samples and not to all qPCR-positive detections. The failure to amplify the *pap31*, *rpoB*, and *groEL* genes of *Bartonella* spp. in this study may be attributed to methodological and biological factors rather than to the absence of the target organism. In particular, the low concentration of bacterial DNA in blood samples—especially in cases of low-level or transient bacteremia—might have limited the efficiency of conventional PCR assays. Additionally, sequence variability among *Bartonella* strains may result in partial or complete mismatches between the primers used and the target regions, thereby impairing amplification. Such limitations are commonly reported in molecular studies of *Bartonella* spp. and should be taken into account when interpreting negative amplification results for these genes, contributing to greater methodological transparency without implying the need for additional experimental procedures.

In a nutshell, this study presents the first molecular evidence of *B. bovis*, ‘*Ca.* M. haemobos’, and *M. wenyonii* infection in cattle from Mozambique, demonstrating that these hemopathogens are circulating in the region. A higher positivity for hemoplasmas (68.9%) was found when compared to that for *Bartonella* spp. (22.1%). Adult cattle appeared to have higher chances of positivity for hemoplasmas. The simultaneous detection of these agents highlights the importance of continuous epidemiological surveillance, especially because subclinical infections may compromise animal productivity and enable the silent maintenance of these pathogens within herds. These findings expand the current understanding of cattle health in Mozambique and provide essential data for developing monitoring, control, and prevention strategies tailored to local conditions. The potential impact of *B. bovis* and hemoplasma infections on the productive performance of beef and dairy cattle should be further investigated.

## Data Availability

All data generated in this study are included in this published article. The sequences of *Bartonella* spp. and hemoplasmas have been deposited in Genbank under accession Nos. PV440601, PV440602, PV440603, PV440604, PV440605, PV440606, PV590298, PV590299 and PV590300.
